# MK-801 treatment affects glycolysis in oligodendrocytes more than in astrocytes and neuronal cells: insights for schizophrenia

**DOI:** 10.3389/fncel.2015.00180

**Published:** 2015-05-12

**Authors:** Paul C. Guest, Keiko Iwata, Takahiro A. Kato, Johann Steiner, Andrea Schmitt, Christoph W. Turck, Daniel Martins-de-Souza

**Affiliations:** ^1^Laboratory of Neuroproteomics, Department of Biochemistry and Tissue Biology, Institute of Biology, University of CampinasCampinas, Brazil; ^2^Research Center for Child Mental Development, University of FukuiFukui, Japan; ^3^Department of Development of Functional Brain Activities, United Graduate School of Child Development, Osaka University–Kanazawa University–Hamamatsu University School of Medicine–Chiba University–University of FukuiFukui, Japan; ^4^Department of Neuropsychiatry, Graduate School of Medical Sciences, Kyushu UniversityFukuoka, Japan; ^5^Innovation Center for Medical Redox Navigation, Kyushu UniversityFukuoka, Japan; ^6^Department of Psychiatry and Psychotherapy–Center for Behavioral Brain Sciences, University of MagdeburgMagdeburg, Germany; ^7^Department of Psychiatry and Psychotherapy, Ludwig-Maximilians UniversityMunich, Germany; ^8^Laboratory of Neurosciences (LIM-27), Institute of Psychiatry, University of São PauloSão Paulo, Brazil; ^9^Department of Translational Research in Psychiatry Proteomics and Biomarkers, Max Planck Institute of PsychiatryMunich, Germany; ^10^UNICAMP’s Neurobiology CenterCampinas, Brazil

**Keywords:** schizophrenia, MK-801, clozapine, neurons, astrocytes, oligodendrocytes, Western blot, glycolysis

## Abstract

Schizophrenia is a debilitating mental disorder, affecting more than 30 million people worldwide. As a multifactorial disease, the underlying causes of schizophrenia require analysis by multiplex methods such as proteomics to allow identification of whole protein networks. Previous post-mortem proteomic studies on brain tissues from schizophrenia patients have demonstrated changes in activation of glycolytic and energy metabolism pathways. However, it is not known whether these changes occur in neurons or in glial cells. To address this question, we treated neuronal, astrocyte, and oligodendrocyte cell lines with the NMDA receptor antagonist MK-801 and measured the levels of six glycolytic enzymes by Western blot analysis. MK-801 acts on the glutamatergic system and has been proposed as a pharmacological means of modeling schizophrenia. Treatment with MK-801 resulted in significant changes in the levels of glycolytic enzymes in all cell types. Most of the differences were found in oligodendrocytes, which had altered levels of hexokinase 1 (HK1), enolase 2 (ENO2), phosphoglycerate kinase (PGK), and phosphoglycerate mutase 1 after acute MK-801 treatment (8 h), and HK1, ENO2, PGK, and triosephosphate isomerase (TPI) following long term treatment (72 h). Addition of the antipsychotic clozapine to the cultures resulted in counter-regulatory effects to the MK-801 treatment by normalizing the levels of ENO2 and PGK in both the acute and long term cultures. In astrocytes, MK-801 affected only aldolase C (ALDOC) under both acute conditions and HK1 and ALDOC following long term treatment, and TPI was the only enzyme affected under long term conditions in the neuronal cells. In conclusion, MK-801 affects glycolysis in oligodendrocytes to a larger extent than neuronal cells and this may be modulated by antipsychotic treatment. Although cell culture studies do not necessarily reflect the *in vivo* pathophysiology and drug effects within the brain, these results suggest that neurons, astrocytes, and oligodendrocytes are affected differently in schizophrenia. Employing in vitro models using neurotransmitter agonists and antagonists may provide new insights about the pathophysiology of schizophrenia which could lead to a novel system for drug discovery.

## Introduction

Schizophrenia is a severe, debilitating mental disorder that directly affects more than 30 million people worldwide (van Os and Kapur, 2009). It is manifested in various forms with symptoms ranging from delusions, hallucinations, and disorganized thoughts to anhedonia, lack of motivation, social withdrawal, and cognitive impairments. The current diagnosis is interview-based and involves communication of subjective symptoms, emotions, and histories between the patient and physician, and categorization of patients is performed using the Diagnostic and Statistical Manual of Mental Disorders 5 (DSM-5) or the International Statistical Classification of Diseases and Related Health Problems 10th revision (WHO, 2010; American Psychiatric Association, 2013). However, these manuals only provide descriptions and qualify the symptoms of psychiatric disorders without providing any neurobiological correlates of the disease (Möller et al., 2015). Therefore, knowledge of the molecular pathways affected in these conditions is still lacking. Furthermore, frequent misdiagnosis occurs since multiple psychiatric disorders can exhibit similar symptoms. For example, symptoms of delusions and depression can occur in schizophrenia, major depressive disorder, and bipolar disorder (WHO, 2010; American Psychiatric Association, 2013). Since it is now recognized as a multifactorial disease with an insidious onset, increasing our understanding of the underlying causes of schizophrenia requires analysis by multiplex methods such as proteomics to allow identification of whole protein networks (Turck et al., 2008). Over the past decade, a number of proteomic studies of *post mortem* brain tissues from schizophrenia patients have been carried out using techniques such as two-dimensional gel electrophoresis and shotgun mass spectrometry. These have resulted in identification of changes in proteins mostly involved in energy metabolism (English et al., 2009, 2011; Martins-de-Souza et al., 2011a), and this is likely to be linked to other observed effects on proteins associated with oxidative stress (English et al., 2009, 2011; Martins-de-Souza et al., 2011a), neuronal structure and transport (Carlino et al., 2011; Chan et al., 2011; English et al., 2011), and cell trafficking and signal transduction (Pennington et al., 2008; English et al., 2011; Föcking et al., 2011; Martins-de-Souza et al., 2011a). Taken together, the changes in these proteins suggest that there is net effect on loss of myelination and synaptic function, leading to dysfunction of specific brain areas, and perturbed networking across distal brain regions (Stephan et al., 2009; Martins-de-Souza, 2010).

Despite these advances in understanding different pathways affected in schizophrenia, it is still not known whether such changes are more prominent in neurons or in specific glial cells. In particular, oligodendrocyte pathology has been reported in several brain regions, whereas no astrocytosis has been detected, leading to the concept that schizophrenia is not a classical neurodegenerative disease (Schmitt et al., 2009). One of the most affected neurotransmitters is the glutamatergic system. A hypofunction of the glutamatergic *N*-methyl-D-aspartate (NMDA) receptor has been proposed to play an important role in the pathophysiology of schizophrenia and recently this receptor became a target of new treatment strategies (Hashimoto et al., 2013; Zink et al., 2014). Dizocilpine (MK-801) is a non-competitive antagonist at the NMDA receptor and has been used as pharmacological model of schizophrenia (Zink et al., 2014). Here, we have addressed this question of cell-specific alterations by acute and long term treatment of neuronal, oligodendrocyte, and astrocyte cell lines with MK-801 and measuring the effects on energy metabolism (Meltzer et al., 2011). This was achieved by Western blot analysis of six enzymes involved in the glycolysis pathway, which we found consistently different in schizophrenia brain tissue (Martins-de-Souza et al., 2011a). It was also of interest to evaluate the potential use of these cell lines and associated biomarker signatures as a novel system for drug discovery in schizophrenia research by investigating the effects of add-on treatment with the antipsychotic drug clozapine on the levels of glycolytic enzymes, since clozapine is known to counteract symptoms produced by MK-801 (Feinstein and Kritzer, 2013).

## Materials and Methods

### Materials

Biochemical reagents were from Sigma–Aldrich (St Louis, MO, USA), unless specified otherwise. The immortalized mouse hippocampal neuronal cell line, HT22 (Li et al., 1997), was a generous gift from Dr. David Schubert (The Salk Institute; La Jolla, CA, USA). The cells were cultured in Dulbecco’s Modified Eagle Medium (DMEM) containing 10% fetal bovine serum (FBS) and differentiated in modified serum-free DMEM, containing 1X N2 supplement, 50 ng/mL nerve growth factor-β, 100 μM phorbol 12,13-dibutyrate, and 100 μM dibutyryl cAMP for 24–48 h before treatment. All treatments were performed in differentiation medium containing 5 ng/mL nerve growth factor-β. Astrocytes (cell line 1321N1; Haedicke et al., 2009) were cultured in DMEM, containing Nutrient Mixture F-12, penicillin (100 units/mL), streptomycin (100 μg/ml) and L-glutamine (2 mM), and 5% FBS. The MO3.13 cell line (Cellutions Incorporated; Burlington, ON, Canada) is an immortalized human cell line with phenotypic characteristics of oligodendrocyte precursor cells (McLaurin et al., 1995; Buntinx et al., 2003). MO3.13 cells were cultured in DMEM containing 10% FBS, penicillin (100 U/mL) and streptomycin (100 μg/mL). To induce an oligodendrocyte phenotype, the cells were treated with 100 nM Phorbol 12-myristate 13-acetate (PMA) for 4 days. All cell lines were cultured at 37°C in 5% CO_2_.

### Cell Culture Treatments with MK-801 and Clozapine

The HT22, 1321N1, and MO3.13 cells were treated with MK-801 and/or clozapine under acute (8 h) and long term (72 h) conditions. For the acute treatment, all cells were treated for 8 h with either vehicle (water), 50 μM MK-801, or 50 μM clozapine. For the combined acute treatment, cells were treated first for 4 h with 50 μM MK-801 and then 50 μM clozapine was added and the incubation continued for another 4 h. The cells were collected and stored at -80°C. In the long-term treatment, cells were treated with either vehicle, 10 μM MK-801, or 10 μM clozapine at 0, 24, and 48 h. For the combined chronic treatment, the cells were incubated as above with 10 μM MK-801 and then 10 μM clozapine was added at 8, 32, and 52 h. Concentrations were chosen based on those of previous studies (Kondziella et al., 2006; Paulson et al., 2007; Martins-de-Souza et al., 2011b). The cells were collected after a total of 72 h and stored at -80°C. All incubations in all experiments were performed three times.

### Western Blot Analysis

Western blot analysis was carried out essentially as described previously (Krishnamurthy et al., 2013). Frozen cell pellets were homogenized in 100 μL of 7 M urea, 2 M thiourea, 4% CHAPS, 2% ASB-14, and 70 mM dithiothreitol (DTT) using a kit for sample grinding (GE Healthcare; Munich, Germany). Protein lysates were centrifuged at 14,000 ×*g* for 10 min. The resulting supernatants were collected and protein concentrations determined using the Bradford assay (BioRad; Munich, Germany). The protein extracts (20 μg) from each cell sample were electrophoresed on 12% sodium dodecyl sulphate (SDS) minigels (BioRad; Hercules, CA, USA). The proteins were then transferred electrophoretically to Immobilon-FL polyvinyldiphenyl fluoride (PVDF) membranes (Millipore; Bedford, MA, USA) at 100 V for 1 h using a cooling system. PVDF membranes containing the transferred proteins were treated with 5% Carnation instant non-fat dry milk powder in Tris buffered saline (pH 7.4) containing 0.1% Tween -20 (TBS-T) for 4 h, rinsed in TBS-T three times for a total of 20 min and incubated with hexokinase 1 (HK1), aldolase C (ALDOC), and enolase 2 (ENO2) antibodies at a 1:2000 dilution and with phosphoglycerate kinase (PGK), phosphoglycerate mutase 1 (PGAM1), and triosephosphate isomerase (TPI) antibodies at a dilution of 1:000 in TBS-T overnight at 4°C (all antibodies were from Abcam; Cambridge, UK). Following the overnight incubation, the membranes were washed twice with TBS-T for 15 min per wash. Next, the membranes were incubated with anti-c-MYC-peroxidase antibody (GE Healthcare; Uppsala, Sweden) for 40 min at room temperature, washed with water and TBS-T, and incubated with Enhanced Chemiluminescence (ECL) solution (GE Healthcare) for 1 min. The membranes were scanned using a Gel Doc^TM^ XR+ System (Silk Scientific Incorporated; Orem, UT, USA) and the optical densities of the immunoreactive bands were measured using Quantity One software (Bio-Rad). Protein loading was determined by staining PVDF membranes with Coomassie Blue R-250 to ensure equal loading in each gel lane.

### Statistical Analysis

In preliminary analyses, Kolmogorov–Smirnov tests were used for all dependent variables to analyze whether there were significant deviations from the normality assumption, which was not the case. Significant differences across groups were determined by analysis of variance (ANOVA) using GraphPad Prism (La Jolla, CA, USA). Differences between groups were determined by *post hoc* analyses using unpaired two-tailed *t*-tests with Welch’s correction. Bonferroni adjustment of the type I error probability was not applied since an adjustment of the error probability would decrease the test power. Due to the explorative study design the findings presented here are not conclusive for a causal relationship.

## Results

### Effects of Treatment of Cultured Cells with MK-801 and Clozapine

#### HT22 Neuronal Cells

**FIGURE 1:**
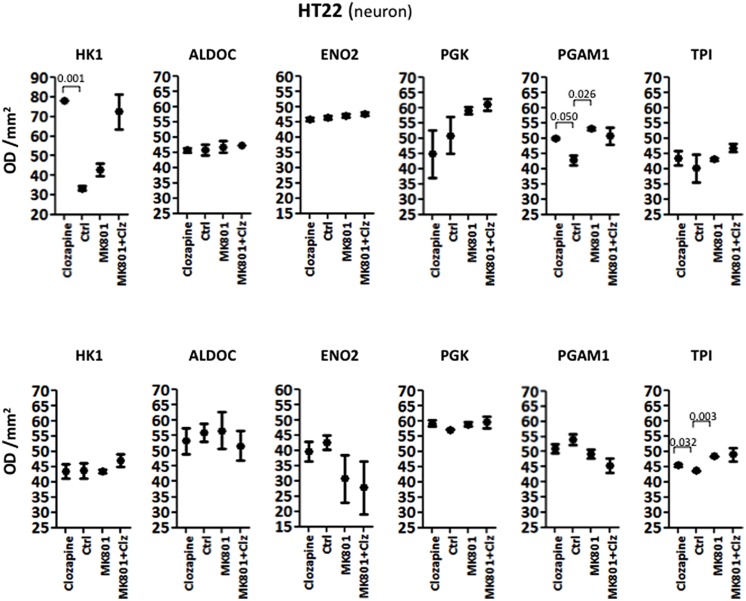
**Levels of glycolytic enzymes in a neuronal cell line (HT22) determined by Western Blot analysis.** The upper panel shows acute effects of MK-801 and Clozapine, and the lower panel shows long-term effects.

Acute treatment of HT22 cells led to significantly increased levels of HK1 and PGAM1, as determined by ANOVA (**Table 1**; see Supplementary Figure S1 for Western blot images of the immunoreactive protein bands for each enzyme under acute and long term conditions in the three cell types). None of the other enzymes showed significant changes. Long term treatment led to a significant increase in the levels of only one enzyme, TPI (**Table 2**). *Post hoc* analysis using unpaired two-tailed *t*-tests with Welch’s correction showed that acute clozapine treatment of the HT22 cells resulted in a marked 2.35-fold increase in HK1 (*P* = 0.001) and a smaller 1.17-fold increase in PGAM1 levels (*P* = 0.050). In addition, acute MK-801 treatment led to a significant 1.24-fold increase in PGAM1 levels (*P* = 0.026). Finally, separate long term treatments with clozapine and MK-801 resulted in respective small but significant increases of 1.04-fold (*P* = 0.032) and 1.11-fold (*P* = 0.003) in the levels of TPI (**Figure 1**).

**Table 1:** Acute treatment of neuronal, astrocyte, and oligodendrocyte cells.

Enzyme	CLOZ	CTRL	MK-801	MK-801/CLOZ	*P*-value
**HT22 cells (neurons)**		
HK1	78.09 ± 0.07	33.29 ± 1.95	42.66 ± 5.57	77.45 ± 15.37	**0.0237**
ALDOC	45.82 ± 1.26	45.87 ± 3.08	46.85 ± 3.42	47.27 ± 0.11	0.4965
ENO2	45.87 ± 0.86	46.48 ± 1.14	47.06 ± 0.96	47.68 ± 0.83	0.1540
PGK	44.82 ± 13.41	51.00 ± 10.60	59.15 ± 2.13	61.08 ± 3.20	0.2875
PGAM1	50.10 ± 0.52	42.81 ± 2.88	53.16 ± 0.73	50.75 ± 4.69	**0.0444**
TPI	43.53 ± 4.05	40.13 ± 8.03	43.15 ± 0.75	46.81 ± 2.31	0.3611
**1321N1 cells (astrocytes)**		
HK1	49.63 ± 2.42	51.25 ± 2.30	49.38 ± 1.67	52.44 ± 1.71	0.3061
ALDOC	52.62 ± 6.90	41.50 ± 2.74	67.30 ± 1.96	62.77 ± 2.41	**0.0156**
ENO2	210.1 ± 1.79	208.3 ± 15.58	200.7 ± 4.16	218.9 ± 0.93	0.1234
PGK	53.74 ± 0.29	49.67 ± 0.40	46.83 ± 1.36	46.50 ± 0.02	**0.0237**
PGAM1	75.92 ± 3.64	76.03 ± 0.89	72.86 ± 3.15	76.04 ± 0.42	0.4415
TPI	214.3 ± 18.13	214.2 ± 45.22	178.0 ± 15.60	278.9 ± 36.73	0.0586
**MO3.13 cells (oligodendrocytes)**		
HK1	55.94 ± 0.09	43.63 ± 1.31	59.90 ± 2.85	51.51 ± 0.25	**0.0156**
ALDOC	51.84 ± 3.87	49.40 ± 2.87	54.32 ± 3.35	60.29 ± 2.60	0.0572
ENO2	337.4 ± 72.78	373.4 ± 13.08	594.4 ± 64.33	235.3 ± 37.45	**0.0307**
PGK	157.4 ± 6.37	155.5 ± 13.11	265.6 ± 7.82	164.4 ± 10.66	0.0770
PGAM1	58.94 ± 0.45	62.17 ± 2.40	55.01 ± 0.02	51.79 ± 1.33	**0.0156**
TPI	76.15 ± 4.95	78.65 ± 6.15	84.60 ± 0.40	80.60 ± 1.40	0.1441

*All cells were treated for 4 h with 50 *μ*M MK-801. After this, 50 mM clozapine (CLOZ) was added and the incubation continued for another 4 h. Cells treated with CLOZ alone were incubated for 8 h. The cells were analyzed by Western blot and densitometric scanning of the immunoreactive bands for the glycolytic enzymes hexokinase 1 (HK1), aldolase C (ALDOC), enolase 2 (ENO2) phosphoglycerate kinase (PGK), phosphoglyceratemutase 1 (PGAM1), and triosphosphateisomerase (TPI). Significantly different treatment groups were detected by ANOVA and *post hoc* analysis was carried out using unpaired two-tailed *t-*tests with Welch’s correction to identify differences between specific groups. *P*-values in bold were significant*.

**Table 2:** Chronic treatment of neuronal, astrocyte, and oligodendrocyte cells.

Enzyme	CLOZ	CTRL	MK-801	MK-801/CLOZ	*P*-value
**HT22 cells (neurons)**		
HK1	43.53 ± 4.05	43.71 ± 4.44	43.48 ± 0.95	47.12 ± 3.62	0.5666
ALDOC	53.28 ± 7.39	55.97 ± 4.85	56.70 ± 10.55	51.71 ± 8.59	0.7888
ENO2	36.69 ± 5.44	42.62 ± 3.98	30.74 ± 13.40	27.84 ± 15.11	0.4243
PGK	59.20 ± 1.78	57.08 ± 0.25	58.91 ± 1.32	59.58 ± 3.26	0.2815
PGAM1	51.03 ± 2.43	54.04 ± 3.08	49.26 ± 2.79	45.36 ± 4.18	0.1129
TPI	45.49 ± 0.73	43.66 ± 0.41	48.42 ± 0.16	48.95 ± 4.03	**0.0434**
**1321N1 cells (astrocytes)**		
HK1	52.91 ± 0.10	52.17 ± 1.08	39.98 ± 0.79	45.33 ± 3.67	**0.0237**
ALDOC	60.98 ± 3.71	59.00 ± 0.71	70.73 ± 3.57	72.09 ± 1.92	**0.0378**
ENO2	180.2 ± 15.30	184.7 ± 16.48	161.5 ± 31.66	151.1 ± 32.02	0.3191
PGK	43.71 ± 0.75	48.56 ± 0.79	48.65 ± 3.50	37.70 ± 2.27	**0.0249**
PGAM1	71.52 ± 0.84	73.14 ± 0.03	72.80 ± 3.50	61.77 ± 3.95	**0.0444**
TPI	186.2 ± 31.83	221.8 ± 45.36	165.6 ± 8.69	322.7 ± 44.19	**0.0378**
**MO3.13 oligodendrocytes**		
HK1	54.67 ± 1.39	50.03 ± 0.08	48.24 ± 0.64	61.73 ± 1.26	**0.0156**
ALDOC	50.92 ± 1.42	52.78 ± 7.10	54.33 ± 0.88	62.63 ± 0.66	**0.0444**
ENO2	409.5 ± 78.89	221.9 ± 10.61	617.6 ± 53.2	229.7 ± 13.22	**0.0237**
PGK	259.0 ± 14.26	158.5 ± 12.36	513.0 ± 13.32	132.0 ± 31.06	**0.0216**
PGAM1	45.32 ± 2.80	45.85 ± 2.07	44.45 ± 4.31	43.00 ± 2.59	0.7152
TPI	78.85 ± 1.25	71.10 ± 0.30	87.95 ± 2.35	92.90 ± 10.20	**0.0237**

*Cells were treated with 10 *μ*M MK-801 at 0, 24, and 48 h and then with 10 *μ*M CLOZ at 8, 32, and 52 h. The cells were collected after a total of 72 h and analyzed by Western blot and densitometric scanning of the immunoreactive bands for the HK1, ALDOC, ENO2, PGK, PGAM1, and TPI. Significantly different treatment groups were detected by ANOVA and *post hoc* analysis was carried out using unpaired two-tailed *t-*tests with Welch’s correction to identify differences between specific groups. *P*-values in bold were significant*.

#### 1321N1 Astrocyte Cells

**FIGURE 2:**
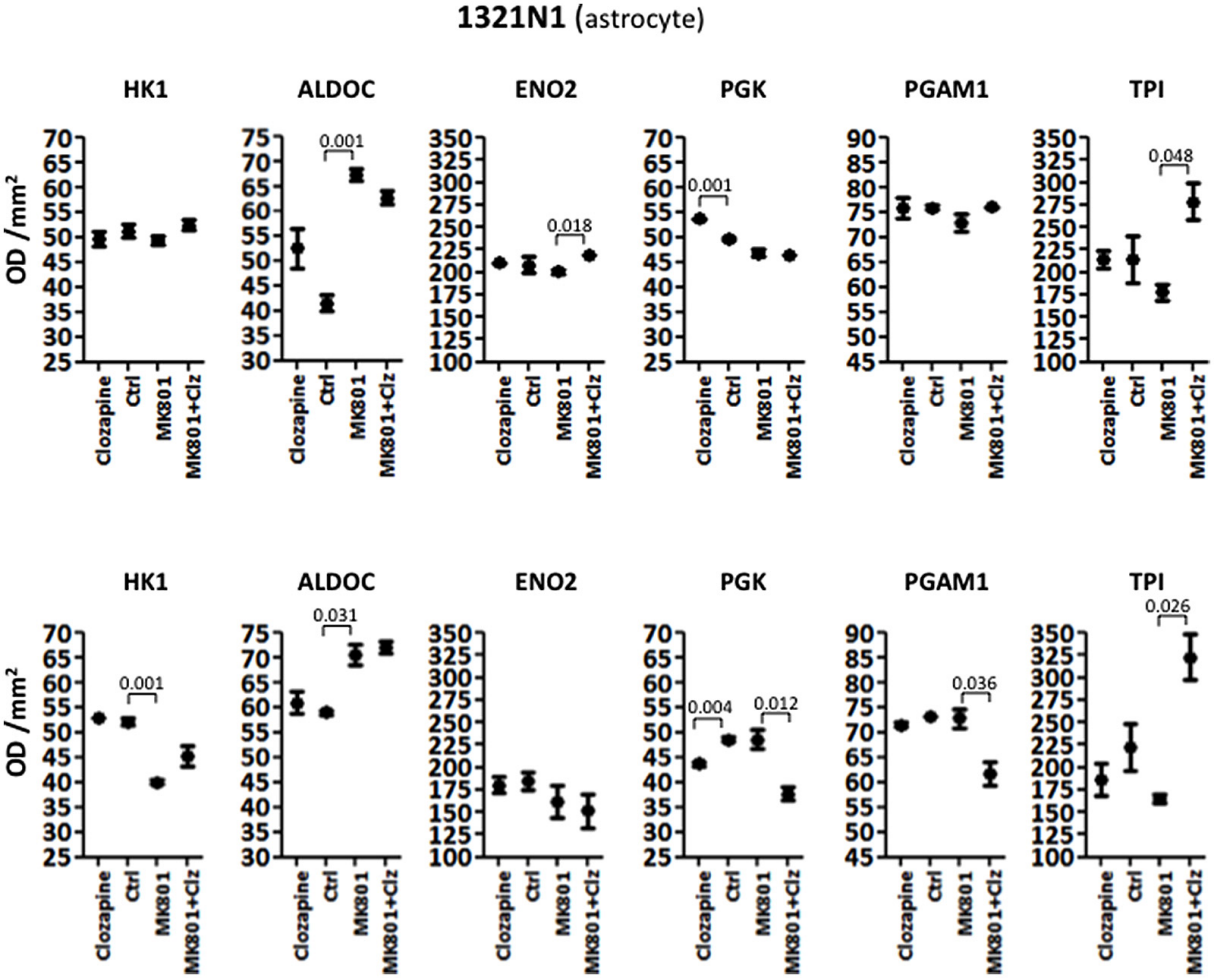
**Levels of glycolytic enzymes in an astrocyte cell line (1321N1) analyzed by Western Blot**. The upper panel represents acute effects of MK-801 and Clozapine, while the lower panel shows long term effects.

Analysis of variance showed that the acute treatment had significant effects on the levels of different enzymes in 1321N1 cells compared with those altered in HT22 cells. In the 1321N1 astrocyte line, ALDOC and PGK were significantly altered (**Table 1**). None of the other enzymes showed significant changes. *Post hoc* testing (unpaired two-tailed *t*-test with Welch’s correction) showed that ALDOC was increased 1.62-fold by acute MK-801 treatment (*P* = 0.001; **Figure 2**). In addition, ENO2 and TPI were increased 1.09-fold (*P* = 0.018) and 1.57-fold (*P* = 0.048), respectively, following the combined MK-801/clozapine treatment in comparison to the treatment with MK-801 alone, and PGK levels were increased 1.08-fold by the clozapine treatment (*P* = 0.001).

Long term treatment of 1321N1 cells resulted in significantly altered levels of five (HK1, ALDOC, PGK, PGAM1, and TPI) out of the six glycolytic enzymes (**Table 2**), as determined by ANOVA. The only enzyme which did not show a change was EN02, which had a non-significant *P*-value of 0.3191. *Post hoc* analysis showed that HK1 was significantly decreased 1.30-fold (*P* = 0.001) and ALDOC increased 1.20-fold (*P* = 0.031) by the long term MK-801 treatment (**Figure 2**). Also, PGK levels were decreased 1.11-fold (*P* = 0.004) by clozapine and 1.29-fold (*P* = 0.012) by the combined MK-801/clozapine treatments compared with the separate MK-801 treatment. Also, PGAM1 levels were significantly decreased 1.18-fold (*P* = 0.036) and TPI levels increased markedly 1.95-fold (*P* = 0.026) by the combined MK-801/clozapine treatment, compared to the MK-801 mono-treatment. Thus, MK-801 had consistent effects on increasing ALDOC levels and the MK-801/clozapine combination led to consistently increased TPI levels relative to the MK-801 treatment alone in both the acute and long term treatment protocols. However, the long term MK-801 treatment led to decreased levels of HK1 and the combined chonic MK-801/clozapine treatment led to decreased PGK and PGAM1, although none of these molecules were affected by the same treatments under acute conditions.

#### MO3.13 Oligodendrocyte Cells

Analysis of variance NOVA analysis showed that the M03.13 cells were affected the greatest by the both the acute and long term treatments, with the highest number of changes in the levels of the glycolytic enzymes tested.

**FIGURE 3:**
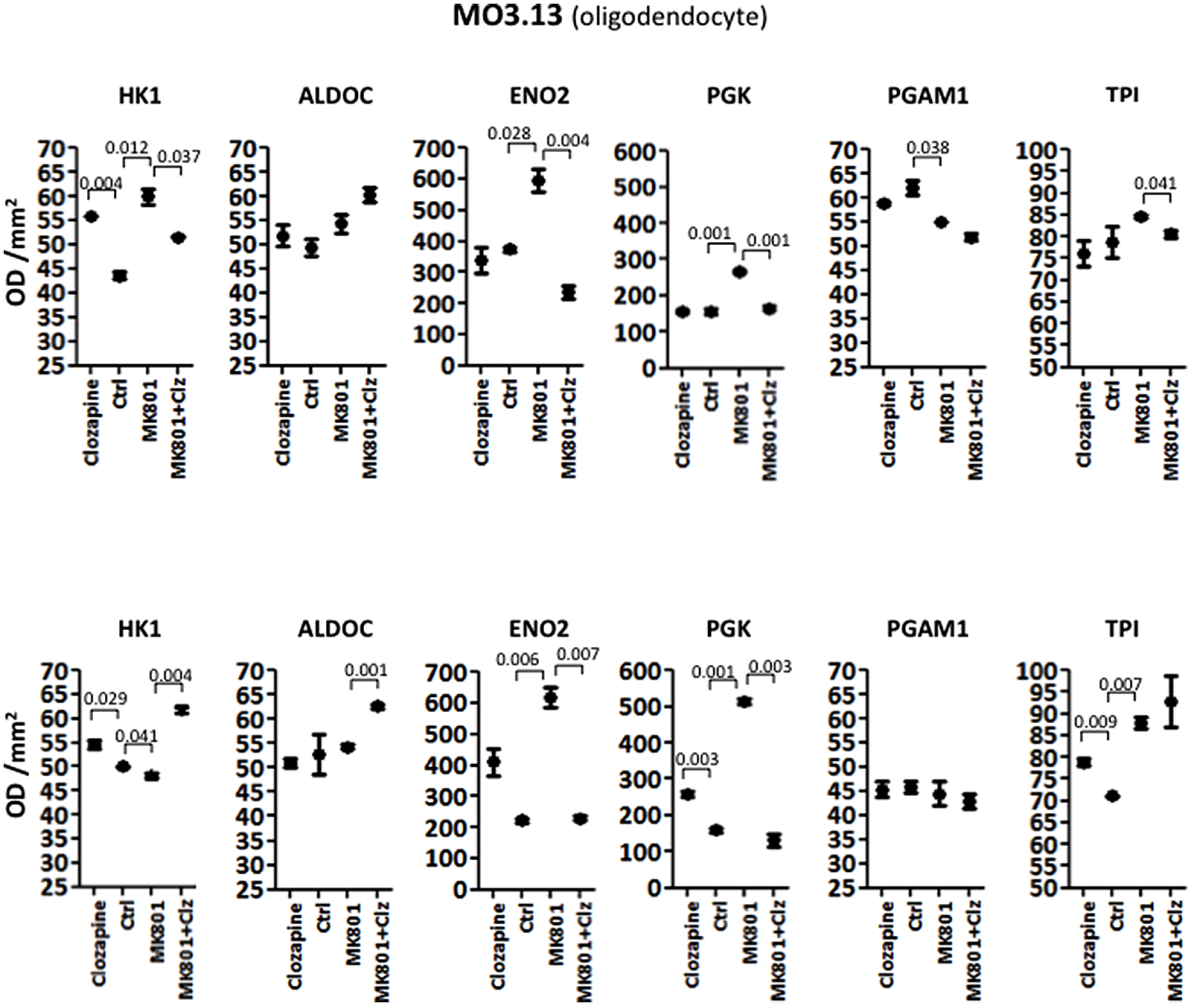
**Levels of glycolytic enzymes in oligodendrocyte cell line (MO3.13) measured by Western Blot analysis**. The upper panel represents acute effects of MK-801 and clozapine treatment, while the lower panel shows long term treatment effects.

The acute treatment resulted in significantly altered levels of HK1, ENO2, and PGAM1. Furthermore ALDOC and PGK showed borderline changes with *P*-values of 0.057 and 0.077, respectively (**Table 1**). Only TPI showed no significant effects (*P* = 0.1441). *Post hoc t*-test analysis, as carried out above, showed that HK1 levels were increased 1.28-fold (*P* = 0.004) by clozapine and 1.38-fold by the MK-801 (*P* = 0.012) treatments, and decreased 1.16-fold by the combined MK-801/clozapine treatment (*P* = 0.037) in comparison to the MK-801 mono-treatment (**Figure 3**). The levels of both ENO2 and PGK were increased markedly by the MK-801 treatment at 1.59-fold (*P* = 0.028) and 1.71-fold and (*P* = 0.001), respectively, and these were decreased to approximately control levels by the combined MK-801/clozapine treatment (*P* = 0.004 and *P* = 0.001, respectively). Finally, PGAM1 levels showed a 1.13-fold decrease (*P* = 0.038) by MK-801 treatment and TPI was decreased 1.05-fold (*P* = 0.041) by the combined MK-801/clozapine treatment compared with the MK-801 mono-treatment.

Long term treatment of M03.13 cells resulted in altered levels of five (HK1, ALDOC, ENO2, PGK, and TPI) out of the six enzymes, as determined by ANOVA (**Table 2**). Four of these (HK1, ALDOC, PGK, and TPI) were changed in common with the 1321N1 astrocyte cells above. PGAM1 was the only enzyme which did not show a significant response to any of the treatments (*P* = 0.7152). *Post hoc* testing showed that HK1 was increased (1.09-fold, *P* = 0.029) by clozapine as found in the acute treatment. However, HK1 levels were affected oppositely in the long term compared to the acute treatment group with a small decrease of 1.04-fold (*P* = 0.041) induced by the MK-801 treatment and a 1.13-fold (*P* = 0.004) increase following the combined MK-801/clozapine treatment compared with MK-801 treatment alone (**Figure 3**). ALDOC was also increased (1.15-fold, *P* = 0.001) by the combined treatment compared to the MK-801 mono-treatment. ENO2 and PGK showed the same pattern as found after the acute treatment with robust increases of 2.78-fold (*P* = 0.006) and 3.24-fold (*P* = 0.001), respectively, following long term treatment with MK-801 and treatment with the MK-801/clozapine combination again led to a decrease approximating the control levels (*P* = 0.007 and 0.003, respectively). However, PGK levels also showed a 1.63-fold increase following the chronic clozapine treatment (*P* = 0.003). Lastly, TPI levels were increased 1.11-fold (*P* = 0.009) by the long-term clozapine treatment and 1.24-fold (*P* = 0.007) by the MK-801 mono-treatment.

## Discussion

This is the first study to show that the main effects on energy metabolism pathways are likely to occur in astrocytes and oligodendrocytes, rather than in neurons, using MK-801 treated cellular models of schizophrenia. Treatment with MK-801 resulted in significant changes in the levels of glycolytic enzymes in all cell types although MO3.13 oligodendrocytes appeared to be the most strongly affected. These cells showed altered levels of four of the enzymes (HK1, ENO2, PGK, and PGAM1) after acute 8 h MK-801 treatment, and the long term 72 h treatment led to similar changes in four of the enzymes (HK1, ENO2, PGK, and TPI). In contrast, the same analysis of the 1321N astrocyte cells showed that MK-801 treatment affected only one enzyme (ALDOC) under acute conditions and two enzymes (HK1 and ALDOC) following long term treatment, and HT22 neuronal cells showed changes in only one enzyme (TPI) following the long term MK-801 treatment protocol. This suggests that astrocytes and neuronal cells are either more resistant to the stresses induced by MK-801 treatment or that oligodendrocytes are more susceptible to treatment with this reagent. It is not clear how these effects are mediated but the findings or recent studies suggest that this could occur through MK-801-induced disruption of glutamate transporters which are associated with mitochondria and energy metabolism enzymes, as found in glial cells and neurons (Domercq et al., 2007; Jackson et al., 2014; Roberts et al., 2014). Considering both the acute and long term treatment protocols, clozapine treatment also had greater effects on the MO3.13 oligodendrocyte cells with changes seen in five out of the six enzymes, 1321N1 cells showed a similar response with changes in four of the enzymes and HT22 cells again showed the lowest response with changes found in only two of the enzymes. Therefore, treatment with the antipsychotic clozapine may have a greater effect on both oligodendrocytes and astrocytes as opposed to neurons. Since all of these cell types possess NMDA receptors which should be susceptible to MK-801 blockade as well as other receptors which are known to mediate the effects of clozapine, the observed differential responses suggest that other systems may be involved. Therefore, further studies aimed at elucidating these differences may lead to identification of novel biomarkers and targets for use in future drug discovery efforts in schizophrenia research.

A previous study using cultured OLN-93 oligodendrocyte cells showed that clozapine treatment improved glucose uptake, as well as the production and release of lactate (Steiner et al., 2014). Under acute conditions in the present study, the MK-801 treatment had only a small effect on the HT22 neuronal cell line in terms of the number of glycolytic enzymes affected. However acute clozapine treatment of these cells led to increased levels of PGAM1. In addition, under chronic conditions, both the MK-801 and clozapine treatments resulted in increased levels of TPI in the neuronal cell line. This suggested that effects on TPI are not likely to be involved in the response to antipsychotic treatment in neurons. It is possible that the observed effect of clozapine on TPI in the neuronal cells is associated with the widely reported metabolic side effects of antipsychotic treatment, such as weight gain and insulin resistance (Deng, 2013). Perturbations of glycolysis are known to be linked with impaired insulin signaling (Belfiore et al., 1979).

Previous studies have found changes in the levels of enzymes involved in glycolysis in brain tissues from rodent models of schizophrenia and from *post mortem* schizophrenia patients. A study of the chronic MK-801 rat model using^13^C-glucose labeling, found reduced glycolysis along with lower glutamate and γ-aminobutyric acid (GABA) levels in multiple brain regions (Eyjolfsson et al., 2011). This demonstrated how reduced supply of glucose or perturbed glycolysis can have effects on the neurotransmitter systems which have been implicated in schizophrenia (Lang et al., 2007). In addition, a proteomic study found that glycolysis was one of the major pathways affected in both gray and white matter areas of *post mortem* brain tissues from schizophrenia patients (Martins-de-Souza et al., 2012a). This is consistent with our finding of effects on the levels of glycolytic enzyme in all three cell types, albeit with more numerous effects in glial cells. One study showed a decrease in HK1 attachment to mitochondria in *post mortem* parietal cortex brain tissue of individuals with schizophrenia which is thought to result in uncoupling of glycolysis with oxidative phosphorylation and, therefore, reduced adenosine triphosphate (ATP) generation (Regenold et al., 2012). HK1 mitochondrial attachment is also thought be important for survival of neuronal cells through prevention of apoptosis and oxidative damage (Saraiva et al., 2010). Again, we found that HK1 levels were affected only in the oligodendrocyte and astrocyte cells after treatment with MK-801. We also found that TPI levels were decreased only in the astrocyte cell line. Martins-de-Souza et al. (2012b) found that the levels of TPI were also decreased in frontal cortex tissue from an acute treatment phencyclidine (PCP) rat model, using selective reaction monitoring (SRM) mass spectrometry in the analysis. The same study also showed that a multivariate signal composed of HK1, ALDOC, ENO2, PGK, PGAM1, TPI, and glyceraldehyde 3-phosphate dehydrogenase (GAPDH) could distinguish the PCP-treated from vehicle-treated control rats using partial least squares discriminant analysis (PLS-DA).

The finding that the oligodendrocyte cells showed a higher number of glycolytic enzyme changes is interesting considering the important role of these cells in myelination of neurons (Nave and Werner, 2014). Numerous lines of evidence have implicated myelin and oligodendrocyte function as critical factors affecting neuronal connectivity, which has also been implicated widely in schizophrenia (Lee et al., 2012; Bernstein et al., 2015; Mighdoll et al., 2015). Therefore, our finding that the MO3.13 cells showed the greatest number of glycolytic enzyme changes in response to the MK-801 treatment is consistent with the possibility that oligodendrocyte pathology and disturbances in white matter tracts may contribute to the pathophysiology of schizophrenia (Schmitt et al., 2009; Yao et al., 2013). Our results suggest that an NMDA receptor hypofunction is associated with oligodendrocyte dysfunction by inducing deficits in glycolysis. However, it remains to be determined whether the effects on glycolysis in these cells are a causative factor or simply a consequence of these processes. Nevertheless, the effects on the glycolytic enzymes in these cells may provide useful biomarkers in drug discovery efforts. In this regard, it is interesting that ENO2 and PGK levels were increased strongly by the MK-801 treatment in the oligodendrocyte cell line and both enzymes were normalized to approximately control levels following treatment with clozapine. Therefore, further studies are warranted to test these two enzymes as antipsychotic treatment response markers in the MO3.13 oligodendrocytes and related cell lines.

### Limitations

Although cell culture studies do not necessarily reflect the *in vivo* pathophysiology and drug effects within the brain, these results suggest that neurons, astrocytes, and oligodendrocytes are affected differently in schizophrenia. Both the acute and long term treatment protocols showed that MK-801 treatment affects glycolysis more in oligodendrocytes than in the other cell types and in some cases these effects could be reversed by antipsychotic treatment. It is not clear whether some of the effects of the antipsychotic treatment are associated with therapeutic efficacy or with the metabolic side-effect profile of these drugs. Further studies are required to address this question. It will also be important to validate the findings by looking for direct changes on mitochondrial structure in the three cell lines. Another set of validation studies will explore if similar changes in glycolysis can be identified in other neuronal or glial cell lines. As a means of validation of these findings, it will also be important to explore if similar changes in glycolysis can be identified in other neuronal or glial cell lines. It will also be important to validate the findings by looking for direct changes on mitochondrial structure in the three cell lines. Finally, the time scale over which the cellular effects occurred in this study is most likely more rapid than that of the disease pathology. We suggest that this is likely to be the case *in vivo* considering that effects at the cellular level precede the systemic effects that eventually lead to the disease and manifestation of symptoms.

## Conclusion

The translation of academic findings to the clinic is now a major objective of biomarker validation studies, especially in support of drug discovery (Owens, 2006; Marson, 2007). We suggest that assays for glycolytic enzymes such as ENO2 and PGK could be implemented on high throughput platforms such as SRM mass spectrometry instruments, considering that this method is robust and user friendly for use in clinical studies. SRM mass spectrometry has already been used in clinical investigations, including screening the levels of dihydroartemisinin (DHA) in plasma of malaria patients (Wiesner et al., 2011), measuring the levels of apolipoproteins in human plasma (Agger et al., 2010) and in cancer biomarker screening studies (Ang and Nice, 2010; Welinder et al., 2014). Finally, the results of the current study indicate that MK-801 treated oligodendrocyte cells may be a useful model for some aspects of metabolic dysfunction and for biomarker screening in schizophrenia studies, considering the known effects on glycolysis-related enzymes in brain tissues from schizophrenia patients. In addition, we propose the use of glycolytic enzymes such as ENO2 and PGK as companion biomarkers for use with this model.

## Author Contributions

PG analyzed the data, researched, wrote, and edited the manuscript. KI carried out the experiments, analyzed the data, wrote, and edited the manuscript. TK conceived experiments, analyzed the data, wrote, and edited the manuscript. JS carried out the experiments, wrote, and edited the manuscript. AS carried out the experiments, wrote, and edited the manuscript. CT researched, wrote, and edited the manuscript. DM conceived and researched the project, analyzed the data, wrote, and edited the manuscript.

## Conflict of Interest Statement

The authors declare that the research was conducted in the absence of any commercial or financial relationships that could be construed as a potential conflict of interest
