# Supplementary material for: MK-801 treatment affects glycolysis in oligodendrocytes more than in astrocytes and neuronal cells: insights for schizophrenia
Source: Front Cell Neurosci. 2015 May 12;9:180. doi: 10.3389/fncel.2015.00180 (PMC4429244; doi:10.3389/fncel.2015.00180)
Supplement: Supplementary file 1 [file Image_1.PDF]

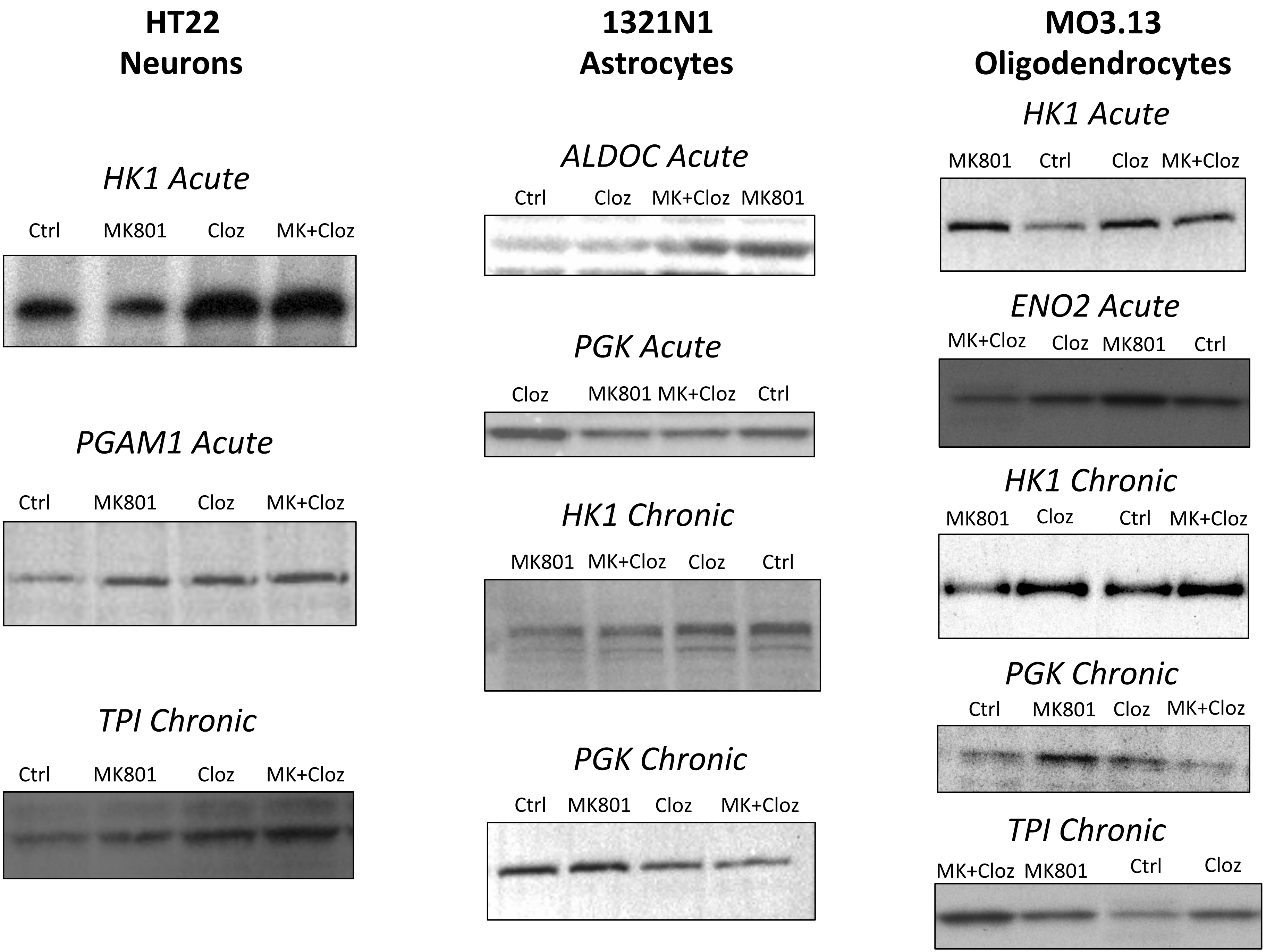

HT22
Neurons
HK1 Acute
Ctrl MK801 Cloz MK+Cloz
PGAM1 Acute
Ctrl MK801 Cloz MK+Cloz
TPI Chronic
Ctrl MK801 Cloz MK+Cloz
1321N1
Astrocytes
ALDOC Acute
Ctrl Cloz MK+Cloz MK801
PGK Acute
Cloz MK801 MK+Cloz Ctrl
HK1 Chronic
MK801 MK+Cloz Cloz Ctrl
PGK Chronic
Ctrl MK801 Cloz MK+Cloz
MO3.13
Oligodendrocytes
HK1 Acute
MK801 Ctrl Cloz MK+Cloz
ENO2 Acute
MK+Cloz Cloz MK801 Ctrl
HK1 Chronic
MK801 Cloz Ctrl MK+Cloz
PGK Chronic
Ctrl MK801 Cloz MK+Cloz
TPI Chronic
MK+Cloz MK801 Ctrl Cloz
